# Closed-chest robotic repair of mitral prolapse. Surgical technique and early results

**DOI:** 10.3389/fcvm.2023.1237151

**Published:** 2023-10-06

**Authors:** Elena Sandoval, Ignacio Morales-Rey, Luis Bartolozzi, Daniel Pereda

**Affiliations:** ^1^ Department of Cardiovascular Surgery, Hospital Clínic, Barcelona, Spain; ^2^ Centro de Investigación Biomédica en Red de Enfermedades Cardiovasculares (CIBERCV), Madrid, Spain

**Keywords:** degenerative mitral valve disease, robotic surgery, thoracoscopic, mini-thoracotomy, surgical times

## Abstract

**Background:**

Robotic mitral repair is generally performed with four intercostal trocars and a minithoracotomy. We describe our technique and results with a totally-thoracoscopic closed chest approach using a 12 mm valveless trocar as “working port”, without a minithoracotomy. We compared our results with this technique with a control group of robotic mitral repairs performed earlier with a minithoracotomy.

**Methods:**

Review of all patients with degenerative mitral valve disease who underwent robotic mitral valve repair surgery since December 2019 (*n* = 110). Patients with concomitant procedures (*n* = 8) were excluded. The remaining 102 patients were divided in two groups, depending on the approach used, minithoracotomy (*n* = 63) and totally thoracoscopic (*n* = 39).

**Results:**

There were no significant differences between groups regarding preoperative characteristics. All procedures were completed robotically as planned, and repair rate was 100%. The minithoracotomy group showed a higher percentage of leaflet resections (17.9% vs. 38.7%; *p* = 0.03). All surgical times were significatively reduced in the totally thoracoscopic group: Cardiopulmonary bypass (97 vs. 115 min, *p* = 0.0008), ischemic time (67 vs. 80 min, *p* = 0.0013) and total surgical time (185 vs. 225 min; *p* < 0.00001). There were no differences in ICU length of stay (1 day, *p* = 0.07) but hospital length of stay was shorter in the totally thoracoscopic group (4 days; *p* = 0.0001). Postoperative complications were similar between groups. MR at discharge was mild or less in all cases.

**Conclusions:**

Robotic mitral repair for degenerative disease can be safely performed as a closed-chest procedure, using a 12 mm trocar as “working port” and avoiding the need for a minithoracotomy. This approach does not seem to negatively affect the quality of the procedure by any measure, providing similar excellent clinical outcomes and repair rate. All surgical times were shorter in the closed-chest group.

## 1. Introduction

Mitral regurgitation (MR) is the most frequent valve dysfunction in developed countries, being mitral prolapse the most frequent etiology of primary MR (1). Surgical mitral valve repair has become the gold-standard treatment for patients with severe MR due mitral prolapse and its indications have expanded in recent years to treat asymptomatic patients with a normal left ventricular (LV) function, provided a successful and durable repair could be expected with low perioperative risk (2, 3).

Over the last 25 years, several minimally-invasive mitral valve surgery (MIMVS) techniques have been developed with the goal of achieving the same or better results while reducing surgical trauma. MIMVS has evolved from a direct-vision procedure performed through a right thoracotomy to a video-directed approach using long-shafted instruments or robotic telemanipulation. However, robotic mitral repair is generally performed with a right minithoracotomy as a working port, that the bedside surgeon uses to assist the console surgeon throughout the procedure (e.g., knot tying, suture tensioning and cutting, tissue traction) and to pass all the materials required in and out of the thorax (e.g., sizers, annuloplasty rings, sutures, resected tissue, debris). A further step to reduce invasiveness would be to eliminate the need for this minithoracotomy and to perform the whole procedure in the closed chest. However, this transition requires several adaptations in both the materials used and the surgical technique.

The aim of this study is to present the technique and initial experience of a totally-thoracoscopic approach for robotic mitral repair, performed exclusively with trocars without a minithoracotomy. We compared the results obtained with this technique with a control cohort of patients operated robotically using a working port.

## 2. Methods

Single-center, observational, retrospective review of prospectively collected data of all consecutive patients who underwent robotic mitral valve repair for degenerative disease in our institution since December 2019–May 2023. Patients with other concomitant procedures (e.g., atrial ablation or tricuspid repair) were excluded. The resulting cohort was divided in two groups, depending on the robotic technique used: minithoracotomy (MT) vs. totally-thoracoscopic (TT).

Descriptive statistics for categorical variables were reported as frequency and percentage, and comparisons were performed using a chi-square test or Fisher's exact test. Continuous variables were reported as median and interquartile range and comparisons were performed using a Wilcoxon-Mann-Whitney test. All comparisons were performed using STATA® (Statacorp LLC; TX, USA).

The study was approved by the Ethics Committee of our institution (HCB 2021/0248) and individual consent was waived.

### 2.1. Preoperative studies

All patients undergo a detailed medical history and physical exam and preoperative thoracic, abdominal and pelvic computed tomography studie to rule out any anatomical issues that may affect the port placement or limit the movement of the robotic arms (scoliosis, pectus excavatum, phrenic nerve palsy and intrathoracic herniation of abdominal viscera, history of prior thoracic surgery, radiation or thoracic trauma) and to evaluate the aortas and the vascular system (arterial and venous). In addition, high-quality comprehensive transthoracic echocardiographic evaluation was performed in all patients and preoperative coronary anatomy was evaluated during CT study or with angiography in selected patients.

Specific contraindications for robotic procedures were:

- Coronary artery disease requiring revascularization.
- Severe peripheral vascular disease or aneurysms of the descending thoracic or abdominal aorta.
- Prior right chest surgery.
- Severe chest wall deformities.
- Ascending aorta dilatation >45 mm or calcification.
- Moderate to severe aortic stenosis or regurgitation.
- Severe calcification of the mitral annulus.

### 2.2. Surgical technique

**Figure 1:**
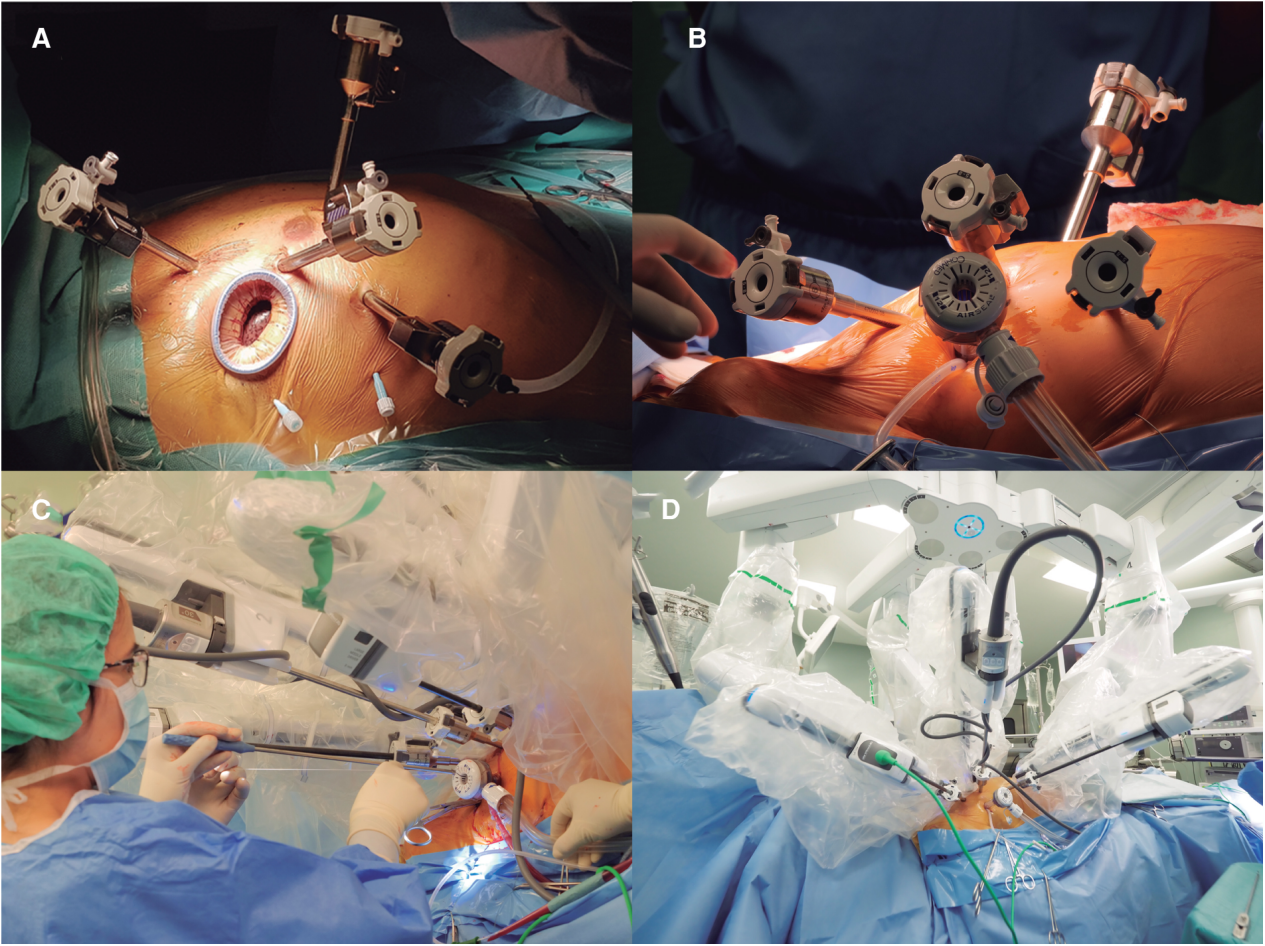
(**A**) Displays the set up for robotic mitral valve repair with the four robotic trocars and a minithoracotomy in the fourth intercostal space. (**B**) Shows the set up used for a totally-thoracoscopic approach where the 12mm trocar replaces the minithoracotomy. (**C**) Shows how the bedside surgeon works using the valveless trocar and (**D**) displays the final set-up for a totally-thoracoscopic approach once the robot is docked.

All procedures were performed with the da Vinci Xi® system (Intuitive Surgical; CA, USA). Patients were placed in supine position with the right chest elevated 30°. Single-lung ventilation was achieved either by using a double-lumen tube or a single-lumen tube and a bronchial blocker. The robotic trocars were placed as follows: the second arm trocar was placed in the fourth right intercostal space near the anterior axillary line and CO_2_ insufflation was connected to obtain a controlled capnothorax of 10mmHg. The camera was then inserted to confirm lung deflation and check for pleural adhesions. Then, under thoracoscopic visualization, 2 guidewires were placed along the posterior axillary line for pericardial retraction sutures. The fourth arm trocar was placed in the sixth intercostal space, slightly posterior to the camera trocar and the first arm trocar was placed in the third intercostal space, in line or slightly anterior to the camera trocar. Finally, the trocar for the third arm was placed in the fifth intercostal space, mid clavicular line (Figure 1A).

In the TT group, a 12 mm AirSeal® valveless trocar (ConMed; FL, USA) was inserted in the fourth intercostal space, 3–4 cm posterior to the camera trocar, to be used as a working port and for continuous CO_2_ insufflation, maintaining the capnothorax pressure throughout the case (Figure 1B). In the MT group, the working port was created by means of a 3–4 cm minithoracotomy in the fourth intercostal space, 2–3 cm posterior to the camera trocar, and a soft tissue retractor is placed (Alexis® XXS. Applied Medical; CA, USA). In the MT group, a third retraction suture is used to retract the diaphragm caudally in most cases, while in the TT group this step was not performed, due to the working space created by the capnothorax in the closed-chest setting.

Simultaneously to the thoracic setup, the anterior wall of the right femoral vessels was exposed with a 3 cm inguinal incision. Before docking the robotic system, heparin was administered, and the femoral vessels were canulated under transesophageal echocardiographic guidance. Once the robot was docked and the instruments inserted (Figures 1C,D), cardiopulmonary bypass (CPB) was initiated. The aorta was clamped transthoracically by the aims of a Chitwood clamp and myocardial protection was performed in both groups using a transthoracic clamp and single-dose crystalloid cardioplegia (Custodiol®. Dr. Franz Köhler Chemie, Germany) was administered in the aortic root through the working port. In both groups the mitral valve was accessed through a left atrial incision and exposed with the aim of the robotic atrial retractor placed in the third arm. Once the valve was exposed and examined using both robotic arms, the repair was performed as described below; “water-test” was used as needed using a flexible tube introduced through the working port.

Once we considered the repair satisfactory, the left atrium was closed using barbed 3/0 V-Loc (Medtronic; MN, USA) sutures, the aortic clamp was removed and the cardioplegia entry site repaired with an automatic suture. We then used the third arm entry site to place a 19F silicone drainage in the pericardial space and the pericardium was loosely closed. The robotic arms were removed, and ventilation restarted. Cardiopulmonary bypass was discontinued, and the repair assessed by transesophageal echocardiography. After assuring a satisfactory repair, the femoral cannulae were removed and protamine administered. Once protamine was administered, careful revision of hemostasis was performed with the camera under single-lung ventilation. Finally, a 24F chest tube was introduced in the right pleural cavity, through the fourth trocar site and all wounds were closed.

**Figure 2:**
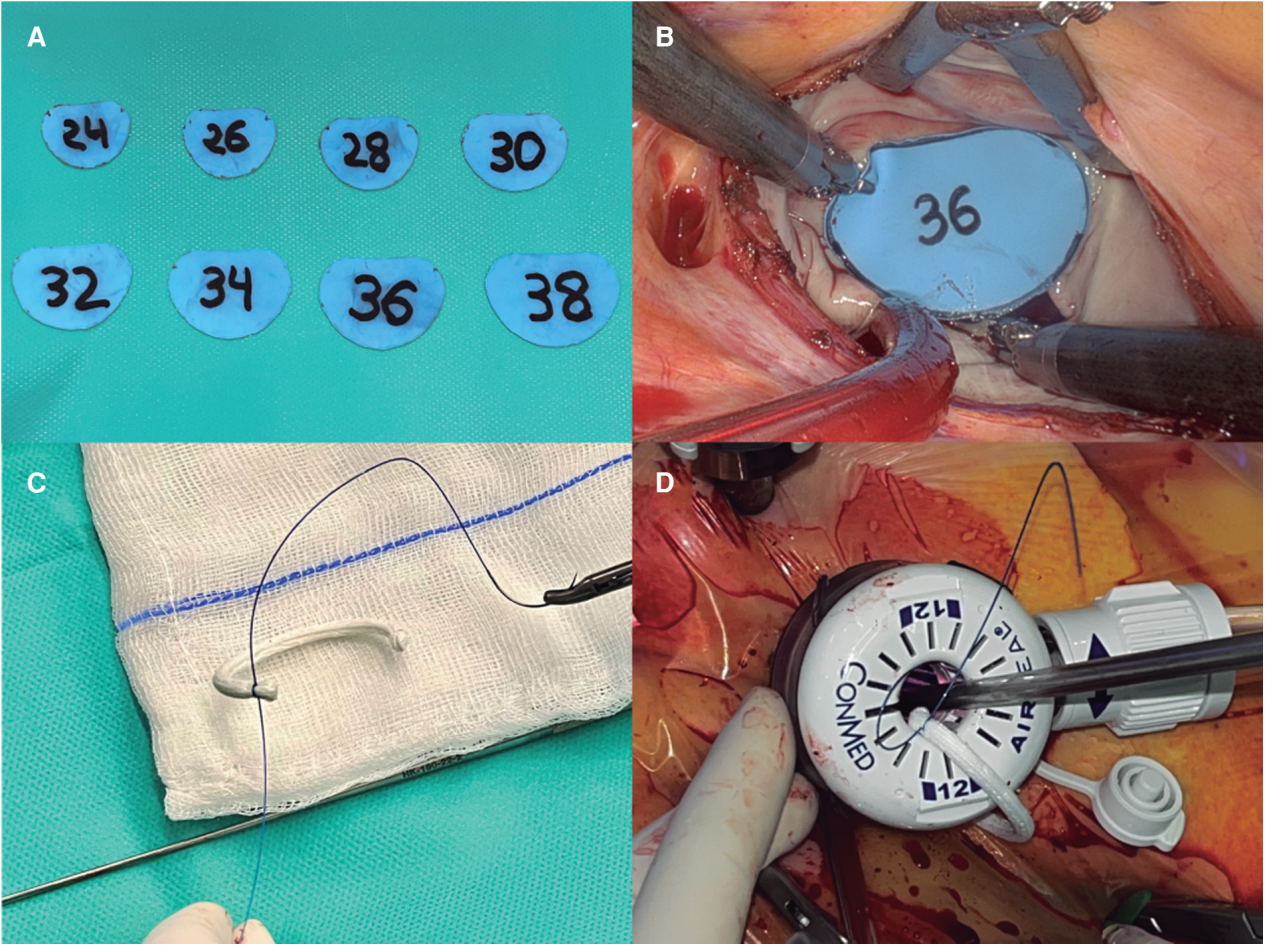
(**A**) Displays the customized annuloplasty sizers, made of flexible material so they can fit through the trocar. (**B**) Shows the sizer covering the anterior mitral leaflet during mitral sizing. (**C**) Shows the band and the barbed suture are prepared and (**D**) demonstrates how the band is entered through the valveless trocar.

In the TT group we have implemented several modifications of the mitral repair procedure to be able to perform the entire operation with the 12 mm trocar. In general, we favor flexible bands for the repair of mitral prolapse (Cosgrove-Edwards annuloplasty system®. Edwards Lifesciences; CA, USA), and they were the only type of annuloplasty device used in the TT group, as they easily fit through the port once removed from the holder (Supplementary Video S1). We also modified the sizing method; instead of using the sizers provided by the manufacturer, we reproduced them in a flexible Esmarck membrane (DeRoyal Industries; TN, USA) that allowed its introduction through the trocar (Figures 2A,B). For the annuloplasty band implantation, we switched from using interrupted sutures secured with the Cor-Knot® device (LSI solutions; NY, USA) to a running suturing technique using two 3/0V-Loc® polybutester sutures (Medtronic; MN, USA) (Figures 2C,D) anchored on both trigones (Supplementary Video S1).

Finally, in the TT group, suture knotting (i.e., neochords, annuloplasty, atriotomy) was performed with the robotic instruments, while in the MT group it was performed by the bedside surgeon using a knot pusher or a Cor-Knot device through the working port.

## 3. Results

Since the beginning of our robotic program in December 2019, 110 patients with severe mitral regurgitation due to degenerative disease were operated robotically. From those, 8 patients were excluded following the exclusion criteria described above. The remaining patients (*n* = 102) were divided in two groups according to the robotic approach used: TT (*n* = 39) and MT (*n* = 63). Since the first totally-thoracoscopic case performed, all subsequent patients were operated using this technique except four: three of them underwent a concomitant Maze procedure and the last one underwent concomitant tricuspid repair; these four patients were therefore excluded. There were no significant differences in baseline characteristics between both groups (Table 1).

**Table 1:** Baseline and preoperative variables.

	Totally thoracoscopic (*n* = 39)	Minithoracotomy (*n* = 63)	*p*-value
Gender (male)	25 (64.1%)	47 (74.6%)	0.26
Age (years)	60.7 +/− 11.1	58.5 +/− 13.7	0.46
Height (cm)	171.1 +/− 9.6	171.1 +/− 9.3	0.96
Weight (kg)	72.3 +/− 12.9	74.4 +/− 15.4	0.41
Hypertension (%)	12 (30.7%)	21 (33.3%)	0.79
Dyslipidemia (%)	6 (15.4%)	15 (23.8%)	0.31
Diabetes mellitus (%)	3 (7.7%)	7 (11.1%)	0.74
LVEF (%)	60 (55–65)	60 (59–65)	0.19
Pulmonary hypertension	10 (25.6%)	16 (25.4%)	0.97
NYHA III–IV	9 (23.1%)	18 (28.6%)	0.54
Preoperative atrial fibrillation	5 (12.8%)	9 (14.3%)	0.83
Cerebrovascular accident	2 (5.1%)	1 (1.6%)	0.56
EuroSCORE II	0.93 (0.62–1.06)	0.95 (0.62–1.72)	0.37
Prolapse - Anterior - Posterior - Bileaflet	3 (7.7%) 30 (76.9%) 6 (15.4%)	5 (7.9%) 40 (63.5%) 18 (28.6%)	0.44

**Figure 3:**
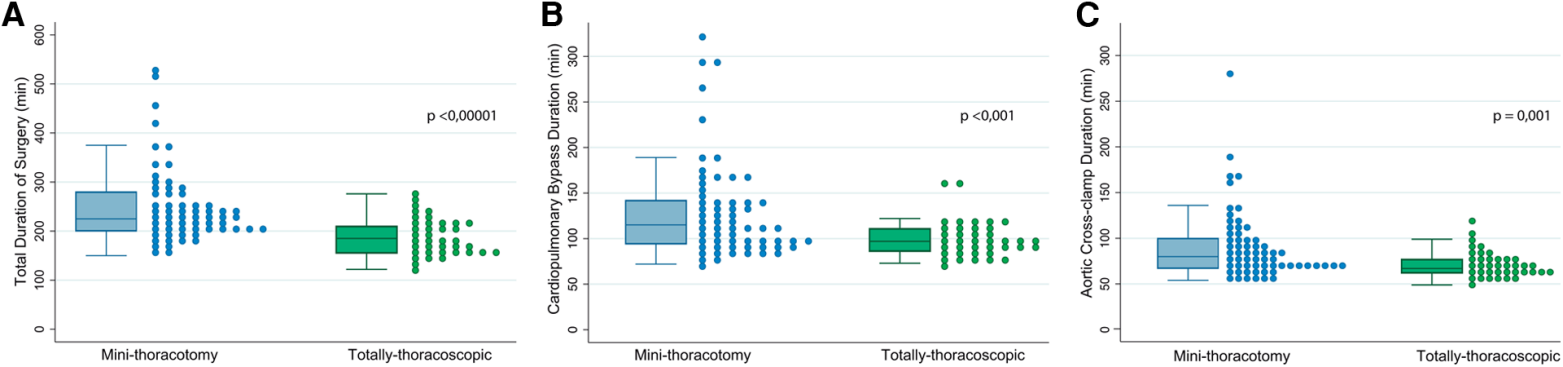
Shows the comparison of surgical times between both groups (MT vs. TT), using both box and scatter plots. (**A**) Corresponds to the total surgical time. (**B**) Shows cardio-pulmonary bypass time and (**C**) presents the durations of ischemic time.

All procedures were successfully completed robotically, without crossover between groups and no conversions to sternotomy. The repair rate was 100% on both groups; the use of neochordae was the most frequent repair technique in both groups (71.8% vs. 68.3%; *p* = 0.71), but the MT group presented a significantly higher rate of triangular resections (17.9% vs. 36.5%; *p* = 0.03). All patients in the TT group received an annuloplasty device, whereas two patients in the MT group did not; one was a patient with hypertrophic obstructive cardiomyopathy and the second one was an 83-year-old patient with preoperative systolic anterior movement. Size 38 mm was the most common annuloplasty size used in both groups (46.2% vs. 49.2%; *p* = 0.56), with 9.5% of patients in the MT group receiving closed semirigid rings. There were no differences in the need for a second cross-clamp time between groups (5.3% vs. 7.9%; *p* = 0.71). All median surgical times were significantly reduced in the TT group compared to the MT group: [CPB: 97 vs. 115 min (*p* < 0.01); ischemic time: 67 vs. 80 min (*p* < 0.01); total surgical time: 185 vs. 225 min (*p* < 0.001)] (Figure 3).

There was neither mortality nor stroke in both groups. Most patients were extubated in the operating room in both groups (76.9% vs. 53.9%; *p* = 0.02). Among those patients not extubated in the OR, a 97.4% were extubated in <12 h in the TT group and 69% in the MT group (*p* = 0.22). The median mechanical ventilation time was similar between groups (6 vs. 8 h; *p* = 0.18). Although transfusion rate was significantly higher in the MT group (10.3 vs. 26.9%; *p* = 0.04), hemoglobin at the third postoperative day was significantly higher in the TT group (105 mg/dl vs. 99 mg/dl; *p* = 0.003). Total chest tube output was significantly higher in the MT (320 ml vs. 570 ml; *p* = 0.006), as it was, despite not significant, reoperation for bleeding (0 vs. 6.3%; *p* = 0.31). All reoperations could be performed through the same approach. Respiratory complications (mainly pneumonia and pneumothorax) were more frequent in the MT group (0 vs. 12.7%, *p* = 0.09). It is also noteworthy that in the MT group, two patients suffered a lesion of the hepatic capsule caused by diaphragm retraction; one case could be solved by interventional radiology while the other one required an open repair. Table 2 describes in detail all postoperative outcomes.

**Table 2:** Perioperative results.

	Totally thoracoscopic (*n* = 39)	Minithoracotomy (*n* = 63)	*p*-value
CPB time (min)	97 (86–111)	115 (94–142)	0.0008
Ischemia time (min)	67 (62–77)	80 (67–100)	0.0013
Total time (min)	185 (155–210)	225 (200–280)	<0.00001
Neochordae	28 (71.8%)	43 (68.3%)	0.71
Type of ring - No ring - Rigid ring - Posterior band	0 0 39 (100%)	2 (3.2%) 6 (9.5%) 55 (87.3%)	0.07
Ring size - 26 - 28 - 30 - 32 - 34 - 36 - 38	0 (0%) 0 (0%) 2 (5.1%) 0 (0%) 9 (23.1%) 10 (25.6%) 18 (46.2%)	2 (3.3%) 1 (1.6%) 1 (1.6%) 3 (4.9%) 9 (14.8%) 15 (24.6%) 30 (49.2%)	0.56
Resection - None - Triangular - Quadrangular	32 (82.1%) 7 (17.9%) 0 (0%)	38 (60.3%) 23 (36.5%) 2 (3.2%)	0.03
Extubation in OR	30 (76.9%)	34 (53.9%)	0.02
Ventilation time (h)	6 (4.5–7)	8 (5–21)	0.18
Drain 24 h (ml)	190 (130–360)	220 (140–360)	0.44
Total drain volume (ml)	320 (205–670)	570 (360–945)	0.006
Hb 3rd day (mg/dl)	105 (95–135)	99 (86–115)	0.003
ICU stay (days)	1 (1–2)	1 (1–3)	0.07
Hospital stay (days)	4 (3–4)	4 (4–6)	0.0001
Complications - Reoperation - Reclamp - Conversion - Atrial fib - Transfusion - CVA - AKI - MR >1 + at discharge	0 (0%) 2 (5.3%) 0 (0%) 7 (18.4%) 4 (10.3%) 0 (0%) 0 (0%) 0 (0%)	4 (6.3%) 5 (7.9%) 0 (0%) 16 (25.4%) 17 (26.9%) 0 (0%) 0 (0%) 1 (1.6%)	0.29 0.71 n/a 0.42 0.04 n/a n/a 0.89

The rate of successful valve repair was 100% in both groups, being MR at discharge mild or less in 100% of the TT group and 98.5% of the MT group. There were no differences in ICU (1 vs. 1 day; *p* = 0.07) stay, but hospital length of stay was significantly lower in the TT group (4 vs. 4 day; *p* = 0.0001). During a median follow-up of 19 months (interquartile range: 9–28), there have been 2 readmissions, both due to Dressler's syndrome. There has been only one reoperation during follow-up, one patient from the TT group who presented recurrent MR during the first year due to reverse ventricular remodeling.

## 4. Discussion

Our study shows that totally-thoracoscopic robotic mitral repair for degenerative disease using a standardized technique is feasible and safe. The TT technique herein described has shown to significantly reduce all surgical times (total operative time, aortic cross-clamp time and CPB time) and hospital stay, as compared to patients operated using a minithoracotomy, without compromising postoperative outcomes or valve repair rate.

The main goal of minimally-invasive surgery is to perform the same surgical procedures, maintaining or improving the quality, while reducing surgical trauma and facilitating patient recovery. In this regard, minimizing or avoiding open incisions in favor of endoscopic alternatives has resulted in better outcomes in multiples areas of surgery in the past, particularly in abdominal and pelvic surgery, but also in thoracic, cardiac and neurosurgical interventions (4–6). Robotic surgery has become in recent years a major advancement in this field thanks to the excellent visualization provided and the improved instrument dexterity, as compared to laparoscopic and thoracoscopic long-shafted instruments. In cardiac surgery, it has become the preferred option for mitral valve repair in several high-volume centers (7–9). Currently, minimally-invasive mitral repair, both in thoracoscopic and robotic cases, is usually performed with the aid of a minithoracotomy that serves as a working port. The minithoracotomy performed may vary significantly in size and is sometimes associated with the use of a rib spreader retractor. Despite some expert centers have been able to evolve the thoracoscopic and robotic techniques to require smaller minithoracotomies and to eliminate the need for rib retraction with good results (6, 10), these procedures are still a minority. We believe that nowadays, with a wide variety of percutaneous treatments competing with surgery, offering less invasive approaches with the same surgical quality is particularly relevant. More so when treating both extremes of the patient spectrum: those in very early stages (asymptomatic, younger, and very active) and those at higher risk (elderly, in advanced stages of the disease or with more comorbidities).

We have evolved our technique in a stepwise fashion with the goal of evolving robotic mitral repair into a fully closed-chest intervention. This objective required eliminating completely the need for a minithoracotomy, and therefore, several adaptations in the materials used and in the surgical technique were developed. First, it required a modification of the sizers since they cannot be introduced through the 12 mm trocar. Second, only flexible bands could be used, introduced with the prosthesis separated from its holder (Figures 2C,D). Third, we changed the annuloplasty technique from interrupted to running sutures, to minimize the number of sutures going through the trocar and the number of knots required. After briefly trying different options, we adopted the technique described in the methods section using barbed sutures, that allowed keeping the tension in the sutures without the need for constant traction by the assistant. This technique was already adopted before switching to the TT approach (in fact, 58.7% of cases in the MT group were performed this way). Finally, all knotting required had to be performed with robotic instruments, with less assistance from the bedside surgeon. This technique has proven even faster for us than using interrupted sutures and Cor-Knot® fasteners (median annuloplasty implant time 17 vs. 30 min; *p* < 0.00001) but requires mindful suture placing and distribution in both the annulus and the band to achieve the desired optimal remodeling.

One of the main concerns regarding minimally-invasive procedures in cardiac surgery is the increase in all surgical times, particularly cross-clamp duration. However, our totally-thoracoscopic technique did not lead to an increase in them; on the contrary, it seemed to allow for faster operations, significantly reducing the ischemic time, the CPB time and the total duration of the intervention. All these procedural times also compare favorably with our Port Access thoracoscopic mitral repair cohort (11). We consider that several factors may have contributed to the total surgical time reduction. First, all robotic cases were performed by the same surgeons, which simplifies the optimization of the new technique and minimizes variability. Secondly, not having to perform a mini-thoracotomy shortens both the entry and the closing time. Thirdly, the continuous capnothorax maintained by the Airseal® trocar provided an excellent surgical field and precluded the need for diaphragmatic retraction sutures, which also speed up the procedure. Ischemic time reduction may have been also influenced by several aspects, one of them being the switch to a running suturing annuloplasty, which greatly reduced the number of sutures tied and shortened the implantation.

A second criticism to less invasive mitral repair is concern regarding safety and the quality of the repair. The incidence of postoperative complications was very low and similar in both groups, with patients in the TT group showing less reoperations for bleeding, transfusion requirements, mechanical ventilation and hospital stay. As mentioned above, all surgical times were significantly reduced in the TT group, including myocardial ischemia. Importantly, repair rate was not compromised by the surgical approach, and was 100% in both groups. Despite the differences seen regarding the use of leaflet resection and chordal replacement techniques in both groups, both were used in the TT approach as deemed appropriate, together with other less common techniques, such as papillary repositioning, chordal shortening/transposition, commissure/indentation closure or edge-to-edge sutures. These findings suggest that the modifications implemented to achieve a closed-chest intervention did not have a negative effect on outcomes.

Some limitations of the present study must be acknowledged. First, this is a retrospective study with the inherent risk of selection bias. In this regard, robotic repair was offered to all patients with degenerative disease who did not present contraindication for robotic mitral valve repair. Furthermore, the TT cohort was completely constituted by consecutive patients, thus minimizing the possibility of selection bias. Second, this study included the learning curve of robotic surgery in our center, which may have unevenly affected patients in both groups, as patients in MT group were operated earlier in the experience. However, to explore this issue, we performed subanalysis after excluding the first 40 patients operated robotically (all located in the MT cohort). In this subanalysis, differences in favor of the TT approach were still significant, including the reduction in ischemic times (67 vs. 73 min; *p* = 0.05) and total surgical time (185 vs. 218 min; *p* = 0.0008). CPB time was also shorter, but the difference did not reach statistical significance (97 vs. 100 min; *p* = 0.14). Thirdly, all robotic procedures were performed by the same surgical team, who has vast experience in minimally-invasive thoracoscopic mitral repair procedures (>500 cases), which may limit the exportability of this results to other environments. Finally, concomitant procedures and other etiologies of MR were excluded and therefore, these results may not be extrapolated directly to procedures other than isolated mitral repair for degenerative disease.

## 5. Conclusions

Robotic mitral valve repair for degenerative disease could be safely performed as a closed-chest procedure, using a 12 mm trocar as a “working port” and completely avoiding the need for a minithoracotomy. This approach did not seem to negatively affect the safety or quality of the procedure by any measure and provides excellent clinical outcomes and repair rates with sorter procedural duration in all its components.

## Data Availability

The raw data supporting the conclusions of this article will be made available by the authors, upon reasonable request.

## Supplementary material

Supplementary Video S1 Displays the totally thoracoscopic repair of an anterior prolapse: The robotic set-up with 4 trocars for the robotic arms in addition to the valveless trocar used as working port can be seen. Once CPB has started, the bedside surgeon places the transthoracic aortic clamp and cardioplegia is infused. Using a left atriotomy, the robotic instruments are used to analyze the valve and, in this case, to resect ruptured chords from the anterior leaflet. After valve analysis is completed, a PTFE suture is entered through the working port and several artificial neochordae are created between the postero-medial papillary muscle and the A3 segment, with the assistance of the bedside surgeon. In addition, the customized sizers enter through the working port and are used to select the proper ring. The flexible band is then prepared, entered through the valveless trocar and implanted with a running barbed suture anchored on both trigones, carefully distributing the sutures to achieve the desired annular remodeling. Once the ring has been implanted, the neochordae are adjusted and tied using the robotic instruments. Valve competency is tested using a “water-test” with a silicone tube entered through the trocar. After ensuring a proper repair, the left atrium is closed with a running barbed suture.
